# National surgical, obstetric, anaesthesia and nursing plan, Nigeria

**DOI:** 10.2471/BLT.20.280297

**Published:** 2021-09-28

**Authors:** Justina O Seyi-Olajide, Jamie E Anderson, Omolara M Williams, Omolara Faboya, Joseph O Amedu, Stanley NC Anyanwu, Abraham Bethuel-Kasimu, Olubunmi A Lawal, Opubo B da Lilly-Tariah, Bisola Onajin-Obembe, Diana L Farmer, Emmanuel A Ameh

**Affiliations:** a Department of Surgery, Lagos University Teaching Hospital, Lagos, Nigeria.; b Department of Surgery, University of Washington, Seattle, United States of America (USA).; c Department of Surgery, Lagos State University College of Medicine, Lagos, Nigeria.; d Federal Ministry of Health, Abuja, Nigeria.; e Department of Surgery, Nnamdi Azikiwe University, Awka, Nigeria.; f National Health Insurance Scheme, Lagos, Nigeria.; g Spina Bifida and Hydrocephalus Care Foundation, Abuja, Nigeria.; h Department of Surgery, University of Port Harcourt, Port Harcourt, Nigeria.; i Department of Anaesthesia, University of Port Harcourt, Port Harcourt, Nigeria.; j Department of Surgery, University of California, Davis, USA.; k Department of Surgery, National Hospital, Central Business District, Abuja, 900001, Nigeria.

## Abstract

Recent evidence suggests that strengthening surgical care within existing health systems will strengthen the overall health-care system. However, Nigeria’s national strategic health development plan 2018–2022 placed little emphasis on surgical care. To address the gap, we worked with professional societies and other partners to develop the national surgical, obstetric, anaesthesia and nursing plan 2019–2023. The aim was to foster actions to prioritize surgical care for the achievement of universal health coverage. In addition to creating a costed strategy to strengthen surgical care, the plan included children’s surgery and nursing: two key aspects that have been neglected in other national surgical plans. Pilot implementation of the plan began in 2020, supported by a nongovernmental organization with experience in surgical care in the region. We have created specific entry points to facilitate the pilot implementation. In the pilot, an electronic surgery registry has been created; personnel are being trained in life support; nurses are being trained in safe perioperative care; biomedical technicians and sterile supplies nurses are being trained in surgical instrument repair and maintenance; and research capacity is being strengthened. In addition, the mainstream media are being mobilized to improve awareness about the plan among policy-makers and the general population. Another development partner is interested in providing support for paediatric surgery, and a children’s hospital is being planned. As funding is a key challenge to full implementation, we need innovative domestic funding strategies to support and sustain implementation.

## Résumé

De récentes preuves suggèrent que le renforcement des soins chirurgicaux au sein des systèmes de santé existants entraînera un renforcement du système tout entier. Pourtant, le plan 2018–2022 de développement stratégique de la santé au Nigeria n'accorde que peu d'importance à ces soins. Pour remédier au problème, nous avons travaillé avec des associations professionnelles ainsi que d'autres partenaires afin de mettre au point le plan national de chirurgie, d'obstétrique, d'anesthésie et de soins infirmiers 2019–2023. Objectif: favoriser les mesures privilégiant les soins chirurgicaux, en vue d'offrir une couverture maladie universelle. Outre l'élaboration d'une stratégie chiffrée servant à consolider le secteur, le plan a intégré les unités de soins infirmiers et de chirurgie pédiatrique, deux aspects clés qui ont été négligés dans d'autres plans nationaux relatifs à la chirurgie. La mise en œuvre de la version pilote du plan a démarré en 2020, avec l'aide d'une organisation non gouvernementale possédant de l'expérience en matière d'interventions chirurgicales dans la région. Nous avons établi des points de départ spécifiques pour faciliter cette mise en œuvre. Dans le cadre de la version pilote, un registre de chirurgie électronique a été créé; le personnel a été formé à l'assistance vitale; les infirmiers ont découvert comment administrer des soins périopératoires sûrs; les techniciens biomédicaux et les infirmiers en stérilisation du matériel ont appris à réparer les instruments chirurgicaux; et enfin, les capacités de recherche et de maintenance ont été revues à la hausse. Par ailleurs, les médias traditionnels ont été sollicités afin d'informer les législateurs et la population en général au sujet du plan. Un partenaire de développement supplémentaire a proposé son aide en matière de chirurgie pédiatrique, et un hôpital pour enfants est prévu. Les fonds constituant l'un des principaux défis d'une mise en œuvre complète, nous avons besoin de stratégies de financement innovantes à l'échelle nationale pour la soutenir et la maintenir.

## Resumen

Las evidencias recientes sugieren que el fortalecimiento de la atención quirúrgica dentro de los sistemas sanitarios existentes reforzará el sistema general de la atención sanitaria. Sin embargo, el plan nacional estratégico para el desarrollo de la salud 2018-2022 de Nigeria dio poca importancia a la atención quirúrgica. Para abordar esta carencia, trabajamos con sociedades profesionales y otros asociados con el fin de elaborar el plan nacional de intervención quirúrgica, obstetricia, anestesia y enfermería 2019-2023. El objetivo era impulsar acciones para priorizar la atención quirúrgica en pro del logro de la cobertura sanitaria universal. Además de crear una estrategia con costes para reforzar la atención quirúrgica, el plan incluía intervenciones quirúrgicas y cuidados de enfermería para niños, que son dos aspectos clave que se han ignorado en otros planes nacionales de intervención quirúrgica. La implementación piloto del plan comenzó en 2020, con el apoyo de una organización no gubernamental que tiene experiencia en la atención quirúrgica en la región. Se han creado puntos iniciales específicos para facilitar la implementación piloto. En el plan piloto, se ha creado un registro electrónico de intervenciones quirúrgicas; se está capacitando al personal en apoyo vital; se está capacitando al personal de enfermería en cuidados perioperatorios seguros; se está capacitando a los técnicos biomédicos y al personal de enfermería de suministros estériles en la restauración de instrumentos quirúrgicos; y se está fortaleciendo la capacidad de mantenimiento e investigación. Además, se está recurriendo a los principales medios de comunicación para dar a conocer el plan a los responsables de formular las políticas y a la población en general. Otro asociado para el desarrollo está interesado en prestar apoyo a la intervención quirúrgica pediátrica, y se está planificando un hospital infantil. Como el financiamiento es un desafío clave para implementar el plan en su totalidad, se requieren estrategias innovadoras de financiamiento nacional para apoyar y sostener la implementación.

## ملخص

تشير الأدلة الحديثة إلى أن تدعيم الرعاية الجراحية في النظم الصحية القائمة سيدعم نظام الرعاية الصحية ككل. ومع ذلك، فقد منحت خطة التنمية الصحية الاستراتيجية الوطنية في نيجيريا للفترة من 2018 إلى 2022 اهتمامًا قليلاً للرعاية الجراحية. ولرأب هذه الفجوة، فقد عملنا مع الجمعيات المهنية والشركاء الآخرين لتطوير الخطة الوطنية للجراحة والتوليد والتخدير والتمريض للفترة من 2019 إلى 2023. كان الهدف هو انتهاج إجراءات لإعطاء الأولوية للرعاية الجراحية لتحقيق التغطية الصحية الشاملة. بالإضافة إلى إنشاء استراتيجية محددة التكلفة لتدعيم الرعاية الجراحية، تضمنت الخطة جراحة الأطفال والتمريض: وهما جانبان رئيسيان تم إهمالهما في الخطط الجراحية الوطنية الأخرى. بدأ التنفيذ التجريبي للخطة في عام 2020، بدعم من منظمة غير حكومية ذات خبرة في الرعاية الجراحية في المنطقة. لقد وضعنا نقاط دخول محددة لتسهيل التنفيذ التجريبي. يتم في المرحلة التجريبية إنشاء سجل جراحي إلكتروني، وتدريب الموظفين على دعم الحياة؛ وتدريب الممرضات على الرعاية الجراحية الآمنة؛ وتدريب تقنيي الطب الحيوي وممرضات الإمدادات المعقمة على إصلاح الأدوات الجراحية، وتدعيم القدرة على الصيانة والبحث. بالإضافة إلى ذلك، يتم تعبئة وسائل الإعلام الرئيسية لتحسين الوعي بالخطة بين واضعي السياسات وعامة السكان. يهتم شريك آخر في التنمية بتقديم الدعم لجراحة الأطفال، ويتم التخطيط لإنشاء مستشفى للأطفال. نظرًا لأن التمويل يمثل تحديًا رئيسيًا للتنفيذ الكامل، فنحن بحاجة إلى استراتيجيات تمويل محلية مبتكرة لدعم التنفيذ واستدامته.

## 摘要

近期有证据表明在现有卫生系统中加强外科护理将有助于加强整个卫生保健系统。但是，尼日利亚的 2018-2022 年国家战略卫生发展计划却没有强调外科护理的重要性。为了弥补这一差距，我们与专业团体和其他合作伙伴合作制定了 2019-2023 年全国性外科、产科、麻醉和护理计划。目的是积极采取行动，提高外科护理在实现全面医疗覆盖方面的优先地位。除了制定加强外科护理的成本策略外，该计划还包括儿童外科和护理（其他国家外科计划中忽略的两个关键方面）。2020 年开始进行该计划的试点实施，由该地区具有外科护理经验的非政府组织提供支持。我们创建了特定切入点以促进该试点实施。试点实施中，创建了电子手术登记处；为全体人员开展生命支持方面的培训；为护士开展围术期安全护理培训；为生物医学技术人员和无菌用品护士开展手术器械维修培训；加强维持和研究能力。除此之外，我们还正在动员主流媒体提高决策者和普通大众对该计划的认识。另一个开发合作伙伴重点关注为小儿外科提供支持，并且正在规划建立一家儿童医院。由于资金是全面实施该计划的关键挑战，我们需要创新的国内资金战略来支持和维持计划的实施。

## Резюме

Согласно последним данным, повышение эффективности хирургической помощи в рамках существующих систем здравоохранения укрепит всю систему здравоохранения. Однако в национальном стратегическом плане развития здравоохранения Нигерии на 2018–2022 гг. хирургической помощи уделяется мало внимания. Для устранения этих недостатков авторы сотрудничали с профессиональными сообществами и другими партнерами, чтобы разработать национальный план в области хирургии, акушерства, анестезии и ухода за больными на 2019–2023 гг. Цель заключалась в поощрении мероприятий, которые уделяют первоочередное внимание вопросам хирургической помощи для достижения всеобщего охвата услугами здравоохранения. Помимо разработки стратегии повышения эффективности хирургической помощи с указанием затрат, план включал в себя детскую хирургию и уход за детьми — два ключевых аспекта, которые не учитывались в других национальных планах в области хирургии. Экспериментальное внедрение плана началось в 2020 году при поддержке неправительственной организации, имеющей опыт оказания хирургической помощи в регионе. Были созданы специальные точки входа для облегчения экспериментального внедрения. Для этого был создан электронный реестр хирургии, персонал обучают реанимационным мероприятиям, медсестер обучают периоперационному ведению больного, биомедицинских работников, а также медсестер, занимающихся стерильными расходными материалами, обучают ремонту хирургических инструментов, а эксплуатационный и исследовательский потенциал усиливается. Кроме того, для повышения осведомленности о плане среди политиков и населения в целом мобилизуются основные средства массовой информации. Еще один партнер в области развития заинтересован в оказании поддержки детской хирургии, поэтому планируется создание детской больницы. Поскольку финансирование является ключевой проблемой на пути полной реализации, нам нужны новаторские стратегии внутреннего финансирования для поддержки и устойчивости реализации.

## Introduction

Surgical conditions account for a larger proportion of the global burden of diseases than previously thought. In a survey of 173 providers and students, surgical conditions were estimated to account for about 30% of the global burden of diseases.^1^ Approximately 17 million deaths per year are potentially avertable by surgery in low- and middle-income countries, with the most cost-effective of all health interventions being essential surgical procedures, including caesarean section, laparotomy and open fracture treatment.^2^ However, initial global efforts to improve access to needed health care in low- and middle-income countries have tended to address medically treatable infectious diseases and excluded surgical care.^1^^,^^2^ Following advocacy by global surgery practitioners, in 2015, the World Health Assembly passed Resolution 68.15 mandating Member States to include emergency and essential surgical care and anaesthesia as a component of universal health coverage (UHC).^3^^,^^4^ A key message from the *Lancet* Commission on Global Surgery in 2015 was that “surgery is an indivisible and indispensable part of health care and that surgical and anaesthesia care should be an integral component of a national health system in countries at all levels of development.”^2^ Global advocates have emphasized the need for countries to have a national strategic plan that specifically addresses surgical care, is designed for their context and developed and owned by stakeholders.^2^^,^^5^

National surgical plans have immense potential to contribute to achieving the sustainable development goals (SDGs).^6^ The framework that has been proposed for the development of national surgical plans is the World Health Organization’s (WHO) six building blocks of a health system.^7^ Evidence has shown that investing in and strengthening surgical care within the existing health-care system would lead to strengthening and improvement of the overall system.^6^^,^^8^ In the United States of America in 2010, the 10 million inpatient operations that were performed accounted for 28.6% of all admissions and operations were performed in every subcategory of the 2010 global burden of disease causes.^9^ Such detailed data are not available in Nigeria, but the findings of that study illustrate how surgical care needs to be integrated across a health system.

Nigeria’s health-care system is organized into a three-tier structure with varying responsibilities at the national (federal), state and local government levels (Box 1).^12^ In 2019, the federal government launched its second national strategic health development plan.^13^ Designed to be implemented between 2018 and 2022, the plan set out national health policy goals and objectives based on the principle of UHC.^14^ Nevertheless, the plan lacks much-needed emphasis on surgical care. In an overview of the essential package of health-care services contained within the strategic health development plan for Nigeria, areas of focus on surgical care were emergency obstetric care, obstetric fistula, eye health and cancers.^14^ Injuries and congenital birth defects which may require surgical intervention accounted for 4.7% and 3.7% of disability-adjusted life years, respectively. Another gap was child and adolescent surgical care. Up to an estimated 85% of children in sub-Saharan Africa will develop a surgical condition by the age of 15 years.^15^ While children younger than 15 years constitute about 42% (89 million) of Nigeria’s estimated population of about 214 million, the review did not consider surgical care in assessing and evaluating the state of child and adolescent health care.^14^

Box 1 **Structure and funding of the health system in Nigeria**

**Structure**

Nigeria’s public health system comprises 27 025 primary-care facilities, 1230 secondary-care facilities and 102 tertiary-care facilities. Although these are the most recent numbers in 2021,^10^ how many of these health facilities are functioning and the level of functionality, particularly in relation to surgical care, are difficult to ascertain.

The Federal Ministry of Health is responsible for policy direction, technical support, international relations on health matters, the national health management information system and the provision of health services at tertiary and teaching hospitals and national laboratories. The health ministries in the 36 federal states and the federal capital territory are responsible for secondary hospitals, regulation and technical support for primary health-care services. Local governments are responsible for provision of primary health care.

The private health sector is an integral part of Nigeria’s health-care system, and private health facilities provide a wide range of services from basic to advanced care. Bidirectional referrals occur between the public and private health sector depending on the capacity of facilities.

**Staffing**

Staffing of the health-care system is structured according to the level of the facility. Primary level facilities are typically staffed by nurses, community health workers, community health extension workers and environmental health officers. Occasionally there may be a medical officer present. Secondary level facilities typically have medical officers, nurses, midwives, laboratory and pharmacy specialists, and community health officers. Tertiary level facilities are staffed by specialist health-care providers.

**Funding**

As in other countries in sub-Saharan Africa, only about one third of all Nigeria’s health funding is from government.^10^ Many low-income and lower-middle-income countries spend less than 6.0% of their GDP on health. Nigeria spent 4.2% (US$ 960 million) of the total budget of US$ 23 billion on health in 2019, which accounts for an estimated 32.1% of the health financing gap in Africa.^10^ Financing of the health-care system in Nigeria is partly supported by international development partners, such as government aid partners in high-income countries as well as international charitable and nongovernmental organizations. Payment for health care in Nigeria is mainly out-of-pocket and a report in 2018 estimated that only about 3.0% of people surveyed had health insurance.^11^

GDP: gross domestic product; US$: United States dollar.

In 2016, before the launch of the national health development plan, the Association of Surgeons of Nigeria, the Nigerian Surgical Research Society and the Nigeria chapter of the American College of Surgeons began working with the federal health ministry to develop a national surgical plan. The aim was to foster actions to prioritize surgical care for the achievement of UHC in Nigeria. In this article we describe the development and implementation of Nigeria’s national surgical, obstetric, anaesthesia and nursing plan 2019–2023, and discuss barriers and solutions to its implementation. 

## Planning

### Baseline assessment

Development of the national surgical plan included a countrywide survey of Nigeria’s existing surgical system in 2018.^16^^–^^18^ We based the assessment on the *Lancet* Commission on Global Surgery’s six core key performance indicators for measuring a health system’s ability to provide safe surgery.^2^ The findings of our baseline survey revealed poor performance in all the global surgery indicators (Table 1), indicating the weak state of Nigeria’s surgical system and the enormity of the tasks involved in strengthening the system to meet the *Lancet* Commission 2030 targets.^19^^,^^20^ Using this framework we were able to define achievable targets for Nigeria’s national surgical plan 2019–2023 (Table 1).

**Table 1:** Baseline assessment and targets for development of Nigeria’s national surgical plan 2019–2023

Core indicator^a^	Definition^a^	*Lancet* Commission 2030 target^a^	Nigeria baseline values, 2018	National surgical plan target 2019–2023
Access to timely essential surgery	Proportion of the population that can access, within 2 hours, a facility that can carry out caesarean delivery, laparotomy and treatment of open fracture (the Bellwether procedures)	100%	Published assessment: 100%^12^ Our assessment: 57.7% of hospitals can be reached by 76–100% of patients within 2 hours	Increase by 40% over pre-implementation value by 2023
Specialist surgical workforce density	Number of specialist surgical, anaesthetic and obstetric physicians who are working, per 100 000 population	20 per 100 000	1.8 per 100 000	Increase density of surgeons, anaesthetists, obstetricians to at least 5 per 100 000 by 2023
Surgical volume	Procedures done in an operating theatre, per 100 000 population per year	5000 per 100 000	58.6 per 100 000	Increase surgical volume by 100% of baseline by 2023
Perioperative mortality tracking	All-cause death rate before discharge in patients who have undergone a procedure in an operating theatre, divided by the total number of procedures, presented as a percentage	100% of countries tracking mortality	NA	Achieve 100% of facilities tracking mortality by 2023
Protection against impoverishing expenditure	Proportion of households protected against impoverishment from direct out-of-pocket payments for surgical and anaesthesia care	100%	35%	Achieve 50–75% of households protected by 2023
Protection against catastrophic expenditure	Proportion of households protected against catastrophic expenditure from direct out-of-pocket payments for surgical and anaesthesia care	100%	36%	Achieve 50–75% of households protected by 2023

NA: not applicable.

^a^ We based the assessment on the core indicators for monitoring of universal access to safe, affordable surgical and anaesthesia care set out by the *Lancet* Commission on Global Surgery, 2015.^2^

Note: Because a comprehensive national survey of the surgical system would be prohibitively costly, in 2018 we carried out a survey of five states in Nigeria, selected by convenience sampling from different geopolitical zones and the federal capital territory.^16^ Assessment was done by volunteer surgical trainees, students, nurses and surgeons who visited the facilities and carried out assessments using the World Health Organization Program in Global Surgery and Social Change surgical assessment tool and a modified tool for assessment of children’s surgical capacity.^17^^,^^18^ Unless otherwise indicated, values for Nigeria are from our baseline assessment.

### Process

We developed the plan following strategic conversations with professional societies and collaboration with the National Postgraduate Medical College of Nigeria, West African College of Surgeons, the Program in Global Surgery and Social Change at Harvard Medical School and the University of California at Davis. We adopted a bottom-up approach to defining Nigeria’s strategic priorities for surgical care, with the involvement of professional societies from surgery, anaesthesia and nursing, under the overall coordination of the federal health ministry. Participants at stakeholder meetings critically evaluated the baseline assessment findings and synthesized them towards formulation of the plan. 

### Objectives and structure 

**Fig. 1:**
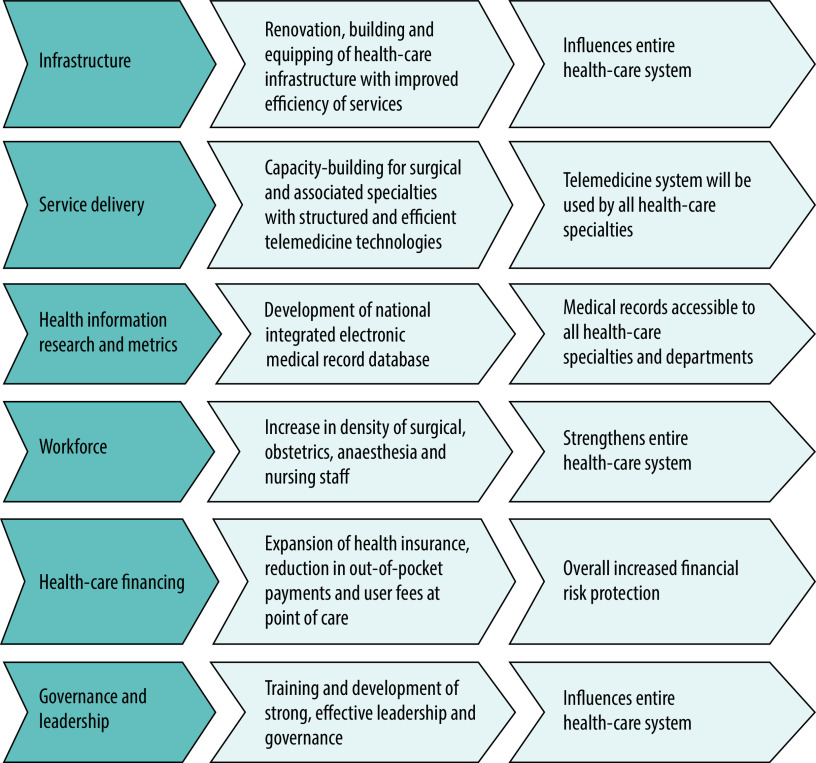
Nigeria’s national surgical, obstetric, anaesthesia and nursing plan for health-system strengthening

Recent progress since 2018 in the delivery of health-related interventions in Nigeria did not necessarily result in gains that were universal or sustainable, especially for the most vulnerable population groups.^11^ Evidence has shown that to achieve improved health status, health systems need to be able to deliver equitable and efficient services.^16^^,^^21^ We therefore designed Nigeria’s national surgical plan around the six building blocks of the WHO health systems framework: (i) infrastructure; (ii) service delivery; (iii) health information, research and metrics; (iv) workforce; (v) health-care financing; and (vi) governance and leadership^22^ (Fig. 1). Using this framework would facilitate integration of the national surgical plan into the existing health-care system. 

Table 2 summarizes the goals and strategies of Nigeria’s national surgical plan within the framework of the WHO building blocks, together with the barriers that need to be addressed. Notable elements of the plan were the priority given to children’s surgery and nursing care, two key aspects that are neglected in other national surgical plans. The rationale for the inclusion of nursing care was that quality nursing care is required for safe surgery and that improvements in surgical outcomes are difficult to achieve without strengthening nursing care. Planned strategies included training and skills acquisition for nurses and an increase in the density of nursing staff. The rationale for the inclusion of children’s surgery was that children account for more than 40% of the population of Nigeria, that surgical conditions are prevalent in children younger than 15 years and that surgical conditions contribute to high mortality in children younger than 5 years.^14^^,^^15^^,^^17^ Our strategies included increasing the workforce for children’s surgery, staff training and the creation of a children’s hospital. 

**Table 2:** Goals, strategies and barriers to achieving Nigeria’s national surgical plan 2019–2023

Health-system building block^a^	Goals	Strategies	Barriers	Solution to barriers
Infrastructure	Strengthen the surgical care infrastructure	Strengthening of existing health-care facilities at all levels Creation of a national children’s hospital	Lack of funding	Deployment of innovative financing solutions
Service delivery	Achieve access to surgical care within 2 hours for 75% of the population Achieve access to surgical care within 2 hours for 50% of children	Expansion of the workforce of surgeons, anaesthetists and obstetricians	Limited training posts Low interest from potential trainees	Increasing the number of training posts Strengthening postgraduate training for the surgical and nursing workforce
Health information, research and metrics	Generate data	Creation of a comprehensive, integrated national electronic health-records database Conducting research	Funding Lack of research capacity	Expansion of research capacity through training
Workforce	Increase the density of surgeons, anaesthetists and obstetricians to 5 per 100 000 population Increase the nursing workforce	Strengthening of postgraduate training Training and supervision of middle-level workforce Task-sharing by delegating tasks to non-specialist providers, with supervision	Limited training posts Low interest from potential trainees	Increasing the number of training posts Strengthening of postgraduate training by the surgical training college Inclusion of funding for postgraduate training in the budget for the plan Strengthening and reorganization of nursing training by the nursing council
Health-care financing	Achieve financial risk protection for 50% of the population	Expansion of public health insurance coverage	Low budget for health care	Deployment of innovative financing solutions
Governance and leadership	Strengthen health care governance at all levels	Application of strategic coordination, supervised by the federal health ministry	Limited awareness of the surgical plan among policy-makers and development partners	Deployment of coordinated and targeted advocacy, including engagement with the media

^a^ We based our framework on the building blocks for health-system strengthening defined by the World Health Organization, 2010.^22^

We prioritized the activities that would need to be implemented over the 5-year period 2019–2023 to achieve the objectives of the plan (Box 2). The implementation of these strategic priorities for surgical care would require the commitment and contribution of all stakeholders, from the government to the general population.

Box 2 **Strategic priorities to achieve the objectives of the national surgical plan, Nigeria, 2019–2023**

**Infrastructure**

- Tracking the number and distribution of surgical facilities
- Equipping first-level surgical facilities (district and general hospitals) to deliver Bellwether procedures: caesarean delivery, laparotomy and treatment of open fractures
- Establishment of a national children’s hospital
- Strengthening of referral systems with community participation

**Service delivery**

- Ensuring all first-level hospitals provide caesarean delivery, laparotomy and treatment of open fractures
- Ensuring provision of safe and quality blood and blood product transfusion services
- Establishment of referral networks, with integration of public, private and nongovernmental organization providers into a common national framework for delivery of services
- Prioritizing quality improvement processes and monitoring of outcomes
- Promotion of telemedicine to build system-wide connectivity to support clinical care and education

**Health information, research and metrics**

- Development of a robust information system to monitor clinical processes, cost and outcomes
- Prioritizing and funding surgical research based on local contexts

**Workforce**

- Establishment of a training and education strategy specific for each state of the country
- Training and education of ancillary staff based on the needs of each state
- Investment in professional training programmes for health-care managers
- Creation of more training programmes for biomedical engineers

**Health-care financing**

- Ensuring basic surgical packages are included within universal health coverage
- Minimizing user fees at the point of care
- Tracking financial flows for surgery through national health accounts
- Ensuring national plan budget allocation at all levels of government health financing

**Governance and leadership**

- Creation of a governance structure that is effective and efficient
- Development of strong and effective leaders to drive policies and performance
- Establishment of a national plan focal person in all relevant ministries, departments and agencies

### Challenges

We encountered several challenges during the development of the national surgical plan. The first challenge was a lack of funding for a countrywide baseline assessment. We therefore scaled down the assessment to one state in each of the six administrative regions (called geopolitical zones) in Nigeria. We also funded the assessment from out-of-pocket spending and accepted the help of volunteers to carry out the assessment and a surgical training college to fund the meeting to draft the plan. The second challenge was the lack of reliable existing data. We undertook primary research and obtained workforce data from medical and nursing councils and the membership databases of surgical and nursing societies. The third challenge was a lack of awareness and commitment by government, development partners and health-care providers. A solution was for the plan to be initiated by Nigeria’s professional surgical societies and the use of advocacy and engagement with the federal health ministry by various professional bodies and development partners.

## Pilot implementation

**Fig. 2:**
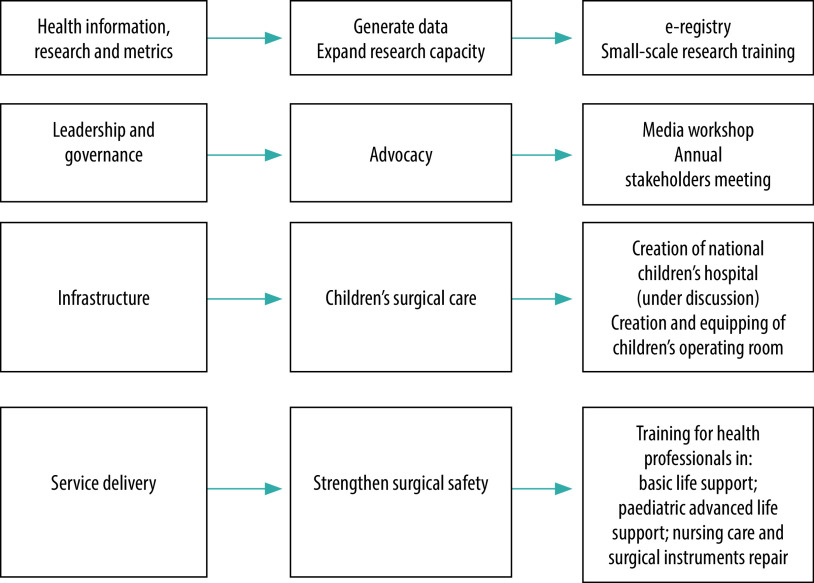
Pilot implementation of Nigeria’s national surgical, obstetric, anaesthesia and nursing plan

Before attempting full-scale implementation, we devised strategies for a pilot implementation of the surgical plan starting in 2020 in collaboration with development partners. To facilitate uptake of specific components by development partners, and to avoid duplication of efforts, we created entry points based on the strategic priorities (Fig. 2). The entry points are small-scale interventions that are easy to fund and implement. The decision about entry points was made by the plan’s implementation team. The entry points also made it possible for government to focus on specific aspects of the plan that could be implemented within the available resources. The following initiatives have been implemented and are gradually being scaled up.

### E-registry

Smile Train is a nongovernmental organization that supports free, comprehensive cleft lip and palate care in low- and middle-income countries.^23^ Smile Train signed a memorandum of understanding with the federal health ministry to develop an e-registry which has been deployed in one institution in one state from each of Nigeria’s six geopolitical zones. The e-registry is an electronic database which is designed to capture details of surgical conditions within the population from identification until after treatment. To facilitate data collection, data collectors and data validators were hired for each institution, with a database manager available to address technical challenges. A statistician provides periodic analysis of registry data. A research assistant and project consultant are available to monitor implementation and provide oversight. All the e-registry personnel have been equipped with customized tablet and laptop computers as well as internet access to facilitate their work. There are regular meetings to track progress, troubleshoot and address challenges. The only challenge identified so far was an inability to upload data, which was quickly resolved by the database manager.

### Life support training

Another priority of the surgical plan was strengthening basic life support skills and paediatric advanced life support (critical care). Training has begun across all six geopolitical zones in the pilot implementation. Twenty-four individuals, consisting of medical officers, trainees as well as consultant surgeons, anaesthetists and paediatricians, were selected from each zone and training took place in one selected location in each zone. Four trainers from the American Heart Association provided training on accredited basic life support and paediatric advanced life support over a period of 6 weeks. A total of 144 doctors have been trained in Nigeria up to September 2021. This number will be increased over the next 5 years. The main challenge encountered has been the small number of trainers in the country. To address the challenge, two trainers delivered training at a time in a particular zone before moving to the next zone so that each set of trainers were involved in three zones. Having identified the shortage of trainers, plans are underway to expand the pool of trainers, by deploying an instructor training for doctors who have undergone the basic and advanced life support training.

### Perioperative nursing care

Smile Train has developed a programme to strengthen perioperative nursing care and improve surgical safety. An initial 24 nurses from the six zones are being trained, beginning with a train-the-trainers programme to create the capacity to deploy training on a large scale. Later, the training will be scaled up to nurses across the country.

### Instrument repair training

Smile Train has also supported the training of biomedical technicians, nurses in the central sterile supplies department and operating room nurses in the repair of surgical instruments involving staff from across the country. An initial 24 participants are being trained and will train other staff for further roll-out of the programme across the country.

### Research capacity training

For the research capacity entry point, we selected 12 surgical and nursing health-care providers from the six zones to undergo a 5-day practical training in the conduct of research, writing grant proposals, and writing and publishing scientific manuscripts. The focus is on early career and mid-level surgical and nursing providers. An earlier 2-day virtual training was deployed for established and senior level surgical and nursing providers to strengthen and update their research skills. The training has been highly successful based on initial feedback. Post-training mentoring is continuing to support the participants and track their success in winning grants and publishing research articles.

### Children’s surgical care

A development partner has taken interest in improving children’s surgical care, and efforts are being made to create a children’s hospital. Given the high capital investment required, completing and deploying the hospital will take several years. The key challenge is raising the required funding. Plans are in place to explore funding options for sustainable maintenance and running of the hospital when it is eventually created. Smile Train is also supporting the creation and equipping of child-specific operating rooms in three tertiary hospitals.

### Media advocacy

Advocacy has played a central role in working towards the future success of the national surgical plan. In April 2021, Smile Train carried out advocacy workshops in three geopolitical zones, targeted at organized media, where the national surgical plan was discussed in-depth with media partners. The aim was to use the organized media to increase awareness among relevant stakeholders about the plan. The media workshop was successful and is already yielding results in terms of a wider understanding among the population and development partners of the importance of surgical care within public health. The key challenge is sustainability of the media advocacy. To overcome this challenge, we have incorporated advocacy into the annual national surgical plan and national cleft week.

## Next steps

The pilot implementation which is still in progress as at September 2021 has not changed the wider plan of implementation of the national surgical plan. However, the impact of the pilot implementation on the wider plan will become clearer over 2022. We are planning to roll out each of the activities in the surgical plan across the country in 2022. The next step will entail a gradual increase in the number of participants in training and increasing the frequency of trainings. To facilitate the increase, we intend to train more instructors so that training can be done in multiple places simultaneously using different training teams.

We are also strengthening regional collaboration. The McGill Centre for Global Surgery in collaboration with the West African College of Surgeons is working to enhance development of surgical plans in West Africa and to support implementation of the plan. The West African College of Surgeons Surgical Plan Committee has created key subcommittees in the areas of advocacy, education, financing and workforce to drive implementation of the national surgical plan across West African countries.

## Barriers to implementation

While steps have been taken by Nigeria’s federal health ministry to begin implementation of the national surgical plan, there are potential challenges to full-scale implementation. Funding for the national surgical plan remains a significant challenge and, currently, no sub-Saharan country has a substantial budget allocation for surgical care. Some of the national surgical plans in other sub-Saharan countries have been costed, ranging from 69.7 million United States dollars (US$) in Rwanda, US$ 171.44 million in Zambia, US$ 597 million in United Republic of Tanzania and US$ 16.8 billion in Nigeria.^24^ The current budgetary allocation to health in Nigeria is low at 4.2% (US$ 960 million) of the total budget of US$ 23 billion in 2019 (Box 1). For Nigeria, 56% of the estimated cost would be for the scale-up of national health-care insurance, which currently covers less than 5% of the population. Given the funding gap, we have proposed innovative financing solutions for implementation of the national surgical plan.^25^ These include expanding the national health insurance scheme, a tax on mobile phone communications, strengthening public–private partnerships as well as leveraging regional in-country investments in West Africa and other parts of the WHO African Region. More advocacy and acceptance is needed by key stakeholders, funders, state and local governments to raise the needed funding for the plan. 

Another challenge has been the coronavirus disease 2019 (COVID-19) pandemic, which required resources and attention to be diverted from other areas of health care. As a result, surgical programmes in Nigeria are at risk of becoming neglected. To avoid this risk, the federal health ministry has emphasized that non-COVID programmes should not suffer during the pandemic. During the peak of the pandemic in March 2020, restrictions on people’s movements as well as COVID-19 protocols in health-care institutions resulted in suspension of elective surgery and anaesthesia, while some surgical and nursing staff were deployed to take care of patients with COVID-19. The already large backlog of surgical care has now become worse. However, surgical care stands to benefit from the investments created by the COVID-19 response, such as the expansion and scaling-up of the intensive care infrastructure and infection prevention and control services in hospitals. These services are important requirements for safe surgical care. Furthermore, non-clinical surgical training activities were successfully moved to internet platforms and have continued online. The successful deployment of online training programmes has shown that such technology could be successfully used to meet workforce training targets in the national surgical plan that do not require the physical presence of trainees.

## Conclusion

Nigeria’s national surgical, obstetric and anaesthesia plan represents a stepwise and organized platform for the strengthening and scaling-up of surgical care in low- and middle-income countries. Funding remains the most important threat to implementation. Continuous innovation of relevant, sustainable funding mechanisms from domestic sources and development partners is required for the implementation and sustainability of the plan. The prioritization of children’s surgery and nursing within Nigeria’s plan strengthens the drive towards UHC as well as supporting the achievement of health-related targets of the SDGs. Implementation of Nigeria’s strategic priorities for surgical care and the achievement of the objectives require the commitment and contribution of all stakeholders including the government and development partners. The successful execution of the plan would transform Nigeria’s health-care system and is attainable despite the challenges inherent in lower-middle-income countries.
